# Genetic control of non-genetic inheritance in mammals: state-of-the-art and perspectives

**DOI:** 10.1007/s00335-020-09841-5

**Published:** 2020-06-11

**Authors:** A. Tomar, R. Teperino

**Affiliations:** 1 Institute of Experimental Genetics, Helmholtz Zentrum München, German Research Center for Environmental Health, Neuherberg, Germany; 2 German Center for Diabetes Research (DZD), Neuherberg, Germany

## Abstract

Thought to be directly and uniquely dependent from genotypes, the ontogeny of individual phenotypes is much more complicated. Individual genetics, environmental exposures, and their interaction are the three main determinants of individual’s phenotype. This picture has been further complicated a decade ago when the Lamarckian theory of acquired inheritance has been rekindled with the discovery of epigenetic inheritance, according to which acquired phenotypes can be transmitted through fertilization and affect phenotypes across generations. The results of Genome-Wide Association Studies have also highlighted a big degree of missing heritability in genetics and have provided hints that not only acquired phenotypes, but also individual’s genotypes affect phenotypes intergenerationally through indirect genetic effects. Here, we review available examples of indirect genetic effects in mammals, what is known of the underlying molecular mechanisms and their potential impact for our understanding of missing heritability, phenotypic variation. and individual disease risk.

## Classical and extended heredity

Years of genetics have attributed uniquely to genes (and genotypes) the ability to generate and transfer phenotypes across generations (Gayon 2016). In 1893, August Weismann in his thesis introduced the theory of heredity where he proposed that in multicellular organisms heritable information is transmitted from germ-plasm (germ cells) to the soma, and this movement is a one way road (Weismann 1893). This theoretical impenetrable barrier is referred to as the Weismann barrier and has blocked till a decade ago any possibility of acquired inheritance since no acquired information can be stored and transferred from the soma to the germline to be inherited (Sabour and Scholer 2012).

Studies from the last decades have broken this dogma and shown that continuous phenotypic traits (such as body mass index—BMI, glucose tolerance, and blood pressure among others) are plastic, respond to environmental challenges during the lifetime and these responses can be inherited across two or more generations, through epigenetic mechanisms (Sabour and Scholer 2012; Skvortsova et al. 2018). This phenomenon, known as epigenetic inheritance, has constituted one of the biggest paradigm shifts in science of the recent years, and extends the classical concept of genetic inheritance to the non-genetic inheritance of acquired characteristics. Phenotypes are thus determined by both genetic and acquired (epigenetic) elements.

## Genome-Wide Association Studies and the Missing Heritability

Genome-Wide Association Studies (GWAS) aimed to identify the genetic basis of human diseases and have provided a genetic framework for our understanding of disease biology, heritability and individual’s susceptibility. While GWAS have identified more than 100,000 strong trait-variant associations (Buniello et al. 2019), many of these variants can only explain a small percent of the observed disease heritability (Manolio et al. 2009). Many indeed failed to predict disease onset in carrier individuals, and could not explain heritability due to phenotypic manifestations in non-carrier offspring of carrier individuals. Known as the “Missing Heritability” problem, this phenomenon highlights a gap in our knowledge of the fundamental mechanisms of phenotypic variation, ontogeny, and inheritance.

The missing heritability is contributed by both genetic and non-genetic phenomena (Bonduriansky and Day 2018; Panzeri and Pospisilik 2018). As a matter of fact, the majority of complex traits are polygenic. Therefore, complex genetic interactions between genes and gene variants associated with the same trait contribute to the phenotype and might explain specific “missing heritability”. To account for this, scientists have proposed a risk scoring system known as polygenic risk score (PRS), which is calculated by weighted sum of risk alleles in an individual and the corresponding effect sizes obtained from GWAS statistics summary, which allows more accurate assessment for individual’s disease risk (Lewis and Vassos 2020). PRS is widely used in neurodegenerative and psychiatric disorders, such as schizophrenia, bipolar disorder and Alzheimer, and can be applied in clinical care to identify individuals at risk and prescribe preventive measures. But studies have identified pitfalls in the PRS construction, which can hinder its practical prediction efficiency, such as lack of diversity in the population included in the studies. Linkage disequilibrium-based pruning for construction of PRS may further lead to bias due to limited reference haplotype panels for various populations. There have been recommendations for reducing the PRS bias in relation to their implementation to populations with varying or admixed ancestries. It is indeed important for GWAS to include diverse populations in order to reduce biases and address health discrepancy (Duncan et al. 2019; Martin et al. 2019; Tam et al. 2019).

The PRS should also consider complex genetic interactions, which affect individual’s phenotypes and might determine the genetic bases of variation in quantitative traits and individual’s risk to complex diseases (Fang et al. 2019; Hill et al. 2008; Sackton and Hartl 2016; Zuk et al. 2012). Epistasis is one example, according to which gene–gene interactions result in masked or altered genotype–phenotype relationships. Undetected epistasis contributes to the missing heritability by overestimating the total heritability of a specific trait and therefore reducing the heritability inferred by GWAS for the same trait—a phenomenon known as “phantom heritability” (Zuk et al. 2012). Genome-Wide Association Interaction Studies (GWAIS) are indeed working towards a systematic identification of statistically significant genetic interactions in GWAS. The major challenges are the complexity of the problem and the associated statistical power which is inversely correlated to the number of identified genetic interactions and hinders discovery (Mackay 2014; Van Steen and Moore 2019). There are multiple epistasis detection tools like BOOST (Wang et al. 2010), EPIBLASTER (Kam-Thong et al. 2011), FRGEpistasis (Franberg et al. 2015; Zhang et al. 2014) which are based on regression-based prediction methods and succeeded in identifying biologically relevant epistasis as shown by studies in model organisms (Costanzo et al. 2016; Mackay 2014, 2015; Zuk et al. 2012). Taking advantage of computational approaches, it is feasible to reveal strong evidence for biologically relevant genetic interactions in various disease contexts (Cordell 2009; Martin et al. 2002). Some studies in model organisms have indeed biologically validated some epistatic signals detected via statistical and computational approaches (Costanzo et al. 2016; Mackay 2014, 2015; Zuk et al. 2012) and in humans, two recent studies have combined statistical and functional approaches to identify functional epistatic interactions in the pathogenesis and risk to coronary artery disease (CAD) (Li et al. 2020) and Scleroderma (Tyler et al. 2020).

Other than polygenic contributions to complex traits and complex genetic interactions (such as epistasis), several additional phenomena have been proposed as potential underlying causes of the missing heritability including intrinsic GWAS experimental limitations, heritability overestimations, variants with small effect-size, epigenetic mechanisms, gene/environment interaction and acquired inheritance, and many others (Lopez-Cortegano and Caballero 2019; Trerotola et al. 2015; Young 2019).

Family studies have also highlighted phenotypic manifestations in wild-type offspring of carrier individuals, thus further contributing to the hindering of the variant-associated heritability score (Bonduriansky and Day 2018). Together with contributing to the missing heritability, this phenomenon highlights the complex, yet critical parental contribution to the offspring phenotypes and disease risk.

## Environmental sensing, phenotypic adaptation, and epigenetic inheritance

**Fig. 1:**
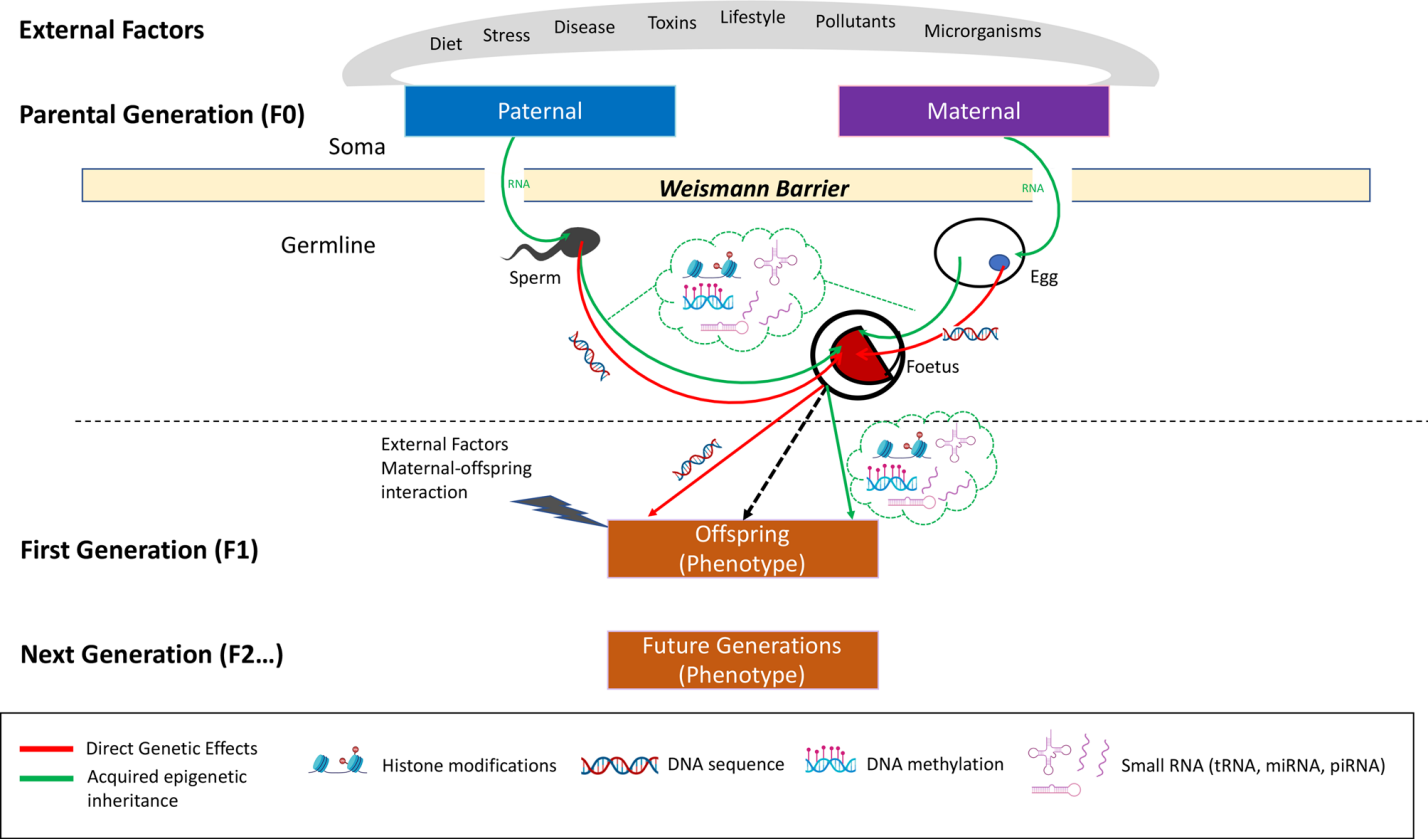
Acquired epigenetic inheritance**.** This figure represents a scheme of the cascade of events characterizing acquired epigenetic inheritance. Phenotypes, acquired by the parental generation via exposure to several environmental challenges, are sensed by the soma and cause epimutations in the germ cells, which, transmitted to the offspring through fertilization, determine their developmental and phenotypic trajectories

Parents can affect offspring phenotypes in different ways. Studies on epigenetic inheritance have shown that parental environmental exposures, either pre-conceptional or during gestation, influence offspring phenotypes across one or several generations (Fig. 1). By definition, if parental effects are only detectable in one non-exposed filial generation (F0 → F1), they are defined as intergenerational. Conversely, should these effects be detectable across multiple non-exposed generations (F0 → F1 → F2 → …), they underlie transgenerational inheritance.

Experimentally, epigenetic inheritance has been described in several model organisms from plants to mammals (David et al. 2019; Hauser et al. 2011; Heard and Martienssen 2014; Holeski et al. 2012; Legoff et al. 2019; Liberman et al. 2019; Lim and Brunet 2013; Opachaloemphan et al. 2018; Perez and Lehner 2019) and it has been mechanistically linked to the acquisition by the developing offspring of environmentally induced epimutations—defined as heritable modifications to the epigenome (e.g., differential DNA methylation, altered chromatin structure, small non-coding RNAs) which induce heritable changes in gene activity without any alteration in the DNA sequence (Oey and Whitelaw 2014)—from the parental germline. Epigenetic inheritance therefore entails two independent phenomena: environmental sensing in the parents, and phenotypic response or adaptation in the offspring. Examples of environmental sensing in the parents followed by inter/transgenerational inheritance have been published in mammals and lower organisms. They underlie the capacity of somatic tissues (including epididymal epithelial cells and neurons) to sense the environment and respond to it and involve inter-tissue and soma-to-germline transfer of small regulatory RNAs (Chen et al. 2016; Conine et al. 2018; Darr et al. 2020; Lev et al. 2019; O'Brien et al. 2020; Posner et al. 2019; Sharma et al. 2016, 2018). Parental driven offspring phenotypic adaptation to environmental stimuli has also been shown as a consequence of epigenetic inheritance. For example, parental exposure to a high-fat diet (HFD) modifies offspring response to the HFD and increase their risk of developing diet-induced obesity and metabolic syndrome (Huypens et al. 2016); paternal exposure to the endocrine disruptor vinclozolin affects offspring fertility and their risk of urological diseases (Anway et al. 2005; Nilsson et al. 2018); and parental brain conditioning by acetophenone exposure coupled to a hot plate stimulus leads to altered brain architecture and response to acetophenone in the offspring (Dias and Ressler 2014). How much of these phenomena can contribute to unravel part of the missing heritability in GWAS remains an open question, but preliminary findings are undoubtedly promising.

The molecular underpinnings of epigenetic inheritance are constantly being unraveled, and current state-of-the-art has been recently and extensively reviewed elsewhere (Skvortsova et al. 2018). For this reason, this review will not focus on epigenetic inheritance of environmentally acquired phenotypes, but introduce the reader to the genetic control of non-genetic inheritance, or the so-called indirect genetic effects in mammals.

## Indirect genetic effects

**Fig. 2:**
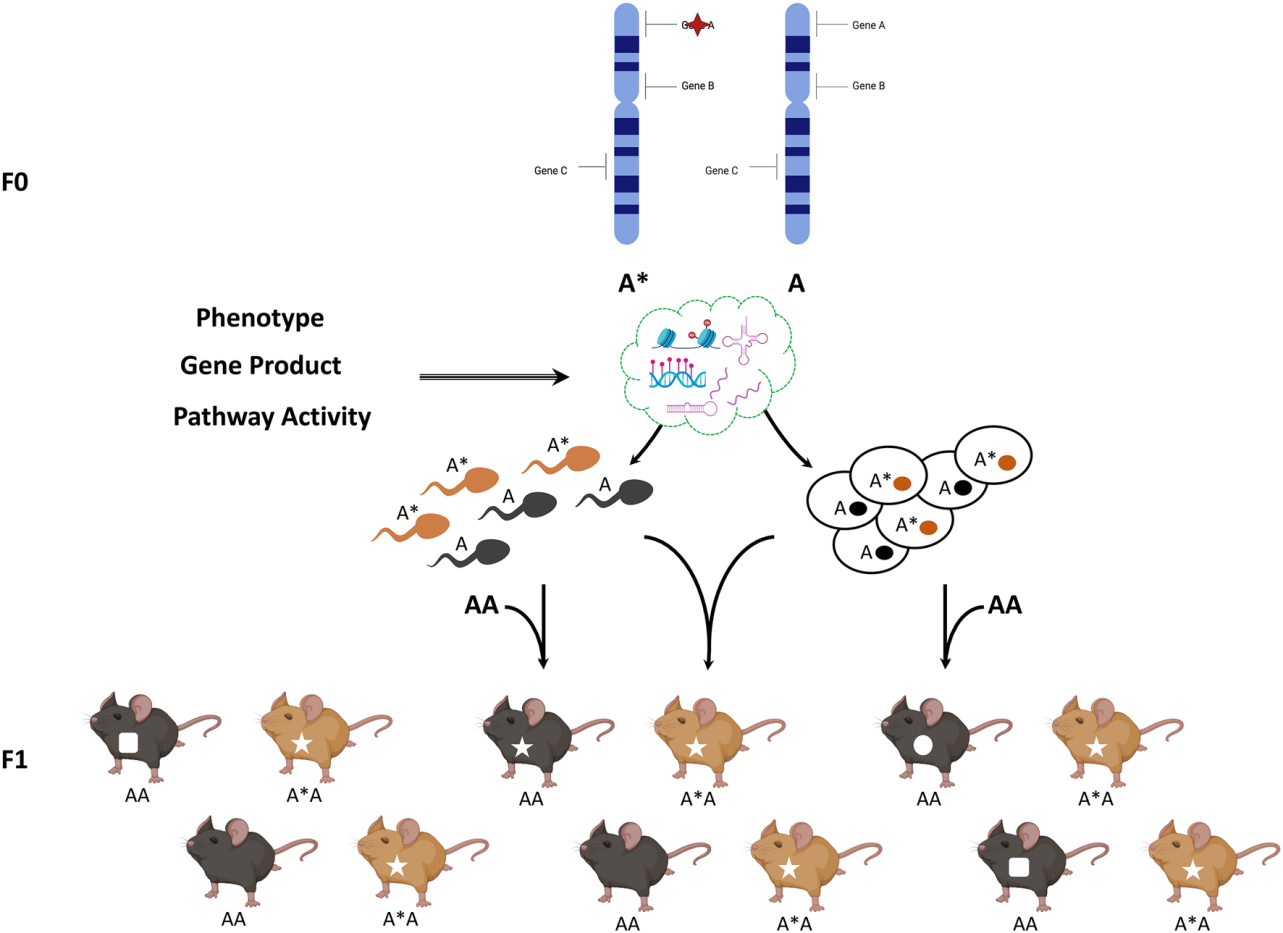
Indirect genetic effects (IGEs). This figure represents a scheme of what we mean by indirect genetic effects. Heterozygous mutations in the parental generation cause—through several and yet undisclosed routes—germline epimutations, which, transmitted to the offspring through fertilization, determine their developmental and phenotypic trajectories independently from the inherited genotype. One interesting feature of IGEs is the increased variability and partial penetrance in offspring phenotypes

Cases where parental genotypes influence the phenotype of non-carrier offspring independently from environmentally acquired phenotypes have been reported (Wolf et al. 1998). Known as Indirect genetic effects (IGEs), these phenomena suggest that specific genetic alterations can reshape offspring phenotypes independently of the inherited genotypes (Fig. 2).

A handful of IGE examples have been reported during the last decades in lower organisms and mammals. In mouse, heterozygous mutations in the Kit-Kit ligand (KitL) system for example lead to a characteristic tail-painting phenotype, which is maintained in wild-type offspring and persists across multiple generations (Rassoulzadegan et al. 2006). Other seminal studies used chromosome substitution strains (CSSs) of mice and showed that the nature of Y-chromosome affects daughters’ phenotypes, with frequency and strength similar to those for conventional inheritance of substituted autosomes (Nelson et al. 2010); or that paternal genotype can control complex phenotypes, such as body weight and food intake, development and heart physiology for multiple generations (Grandjean et al. 2009; Wagner et al. 2008; Yazbek et al. 2010). Yazbek et al., in particular, used the obesity-resistant C57BL6/J congenic strain 6C2d and showed that the paternal or grand-paternal presence of the obesity resistance QTL (Quantitative Trait Locus) *Obrq2aA/J* is sufficient to provide resistance to diet-induced obesity and inhibits food intake in the C57BL6/J progeny. Indirect genetic effects have also been implicated in the parental control of offspring risk to develop cancer. In particular, parental mutations in genes involved in the RNA editing pathway, such as the cytidine deaminase Apobec1 (ApoB editing complex 1) (Nelson et al. 2012) and the RNA-binding proteins Dnd1 (Deadend1) (Lam et al. 2007; Nelson et al. 2012), A1cf (APOBEC1 complementation factor) and Ago2 (Argonaute2) (Carouge et al. 2016) modify offspring’ risk of testicular diseases, including atrophy, cryptorchidism, and testicular germ cells tumor in a genotype and parent-of-origin specific manner.

Canonical epigenetic modifiers, such as DNA methyl transferases (Chong et al. 2007), histone methyl transferases (Prokopuk et al. 2018; Stringer et al. 2018) and demethylases (Lesch et al. 2019; Siklenka et al. 2015) and chromatin remodelers (Chong et al. 2007) have also been shown to induce IGEs in mice. In particular, the group of Emma Whitelaw developed a reporter system featuring variegating expression of a Green Fluorescent Protein (GFP) under the control of an erythroid specific promoter (thus detectable at single cell resolution and in live animals) and coupled it to a ENU (N-ethyl-N-nitrosourea) mutagenesis dominant screen to identify epigenetic suppressors or enhancers of variegation (Blewitt et al. 2005). Interestingly, from the screen they also isolated the chromatin remodeler Smarca5 and the DNA methyl transferase Dnmt1, whose paternal mutations modify the expression of a maternally inherited metastable Agouti-A^vy^ epiallele in wild-type offspring, strongly indicating that the untransmitted parental genotype (in this specific case paternal) can influence the phenotype of wild-type offspring (Chong et al. 2007). Following this study, the group of Sarah Kimmins reported that testis-specific overexpression of the histone demethylase LSD1 (lysine specific demethylase 1) severely impairs development and survivability of wild-type offspring across several generations. Mechanistically, LSD1 overexpression is associated with changes in RNA profiles in the parental germline and offspring tissues and is independent from changes in DNA methylation at CpG sites (Siklenka et al. 2015). Along the same line, the group of David Page showed that perturbing H3K27 methylation in the male germline, by knocking out the H3K27-specific demethylase KDM6a (Utx), increases cancer incidence in wild-type offspring (Lesch et al. 2019). This phenotype becomes stronger following two passages across a mutant germline, but is lost following a single passage through a wild-type germline, interestingly suggesting that epimutations induced by Utx deletion are transmitted to the wild-type progeny and erased during spermatogenesis. Mechanistically, perturbation of H3K27 methylation seems to affect locus-specific DNA methylation, which, partially maintained in the wild-type offspring, leads to phenotype-associated transcriptional differences in target tissues (Lesch et al. 2019). Notably, perturbation of either H3K4 (Siklenka et al. 2015) or H3K27 (Lesch et al. 2019) methylation in the paternal germline leads to phenotypic alterations with increased variation in the wild-type offspring, such as an array of developmental phenotypes or incidence of several tumor types, respectively. These findings thus add further evidence to the notion that complex phenotypes are continuous and “metastable” and that epigenetic mechanisms are critical buffering systems whose alterations can increase variability and phenotype triggering within and across generations (Dalgaard et al. 2016; Panzeri and Pospisilik 2018). More findings support the involvement of parental H3K27 methylation in IGEs and its importance for offspring health. In particular, the group of Patrick Western reported that an ENU-induced Eed (critical structural component of the Polycomb Repressive Complex 2, responsible for H3K27 methylation) hypomorphic mutation in the paternal germline leads to altered developmental timing in wild-type offspring, which is associated with transcriptional deregulation of transposable elements (in particular LINE elements) and retrotransposed pseudogenes in fetal male germ cells and early embryo, respectively (Stringer et al. 2018). The same group has also shown that specific knockout of EZH2 in the maternal germline leads to intergenerational overgrowth (Prokopuk et al. 2018) evident in heterozygous offspring of homozygous mothers compared to heterozygous offspring of heterozygous mothers.

All together, these findings provide evidence that:

1. indirect genetic effects exist in mammals;
2. they can be induced by parental perturbation of both canonical epigenetic modifiers (Smarca5, Dnmt1, Lsd1, Utx, Eed, Ezh2) and genes with previously unknown epigenetic function (Kit, Y-chromosome associated genes, Obrq2a, Apobec1, Dnd1, A1cf, Ago2), and
3. they generally persist across several generations.

Interestingly enough, indirect genetic effects have been also identified in two large humans studies. Kong et al. involved more than 20,000 probands and their genotyped parents to look at the effect of transmitted and non-transmitted parental genetic information on offspring educational attainment (Kong et al. 2018). The findings indicate that non-transmitted parental alleles have an estimated effect on the offspring educational attainment that is 30% of that of transmitted parental alleles. Interestingly, the authors obtained similar results by looking at other complex phenotypes, such as body mass index (BMI), fasting glucose levels and high-density lipoprotein cholesterol (HDL) among others, for which maternal effects are significantly stronger than paternal, despite both being significantly associated with offspring phenotypes (Kong et al. 2018). Along the same line, Bennett et al. enrolled 1316 families with members affected by type 1 diabetes to study the effect of the INS VNTR alleles on disease incidence. What they interestingly found is that the pathogenicity of the risk class I allele was prevented when the same allele was inherited from fathers heterozygotes for the class I risk allele and the class III protective allele, strongly suggesting that the untransmitted class III allele was determining offspring phenotypes despite the presence and inheritance of the class I allele (Bennett et al. 1997).

These studies show that individual’s phenotypes—while mostly contributed by direct inheritance of genetic elements from the parents—are also substantially influenced by non-genetic mechanisms, such as gene/environment interaction, epigenetic inheritance and indirect genetic effects. Therefore, while treated for long by empirical scientists as an annoyance to be statistically controlled, these phenomena can directly influence evolution as well as heritability and its calculation and therefore can complicate predictions of individual’s disease risk (Bijma 2014).

## Paramutations

Despite a growing body of evidence in support of indirect genetic effects, the findings remain mostly descriptive and the underlying molecular mechanisms are far from being completely understood. Kong et al. renamed the indirect genetic effect identified in their human study as “*genetic nurture*” to indicate that parental genotypes (and genotypes of people sharing the same environment) influence the living environment and thereby shape the phenotype of non-carrier individuals (offspring or siblings) sharing the same environment. This is a likely explanation for human studies, where—for the majority of them—it is impossible to avoid parent/offspring environmental sharing, and very hard to dissect molecular mechanisms. Conversely, similar studies in experimental models offer the opportunity to control for developmental and post-natal environment and dissect (or try to) the underlying molecular mechanisms.

In principle, and similar to acquired epigenetic inheritance, parental genetic alterations could induce germline epimutations that segregate independently from the mutated allele and lead to phenotypic manifestations in wild-type offspring (Fig. 2). One interesting mechanistic hypothesis involves *paramutations-like phenomena*. A paramutation is one example of genetically controlled heritable epigenetic variation which defies Mendel’s first law of inheritance, according to which alleles are transmitted unchanged. The first example of paramutation was reported independently by the studies of Brink (Brink 1956) and Coe (Coe 1959) on the *r1* and *b1* loci in maize, whose results—at odds with Mendelian rules—were first dismissed as a curiosity with limited significance, before laying the foundations for any future study on paramutation.

The basic tenet of paramutation is trans-homologous interactions between alleles namely *paramutagenic* and *paramutant*. The *paramutagenic* allele in heterozygotes transmits the phenotype to the wild-type allele (“paramutant”) in a manner which is maintained through multiple generations. A universal hallmark of paramutation is that paramutant alleles become paramutagenic following exposure to another paramutagenic allele in *trans* (Brink et al. 1960; Brown and Brink 1960; Coe 1959; Goettel and Messing 2013; Hollick et al. 1995; Sidorenko and Peterson 2001)*.* In other words, these alleles are metastable. Thus, paramutation can be identified as heritable epigenetic programming of one allele by the other in the same locus. As a result of allelic interaction, the paramutant allele may present with different DNA methylation and/or histone modification patterns, which effect gene expression. Although not yet completely characterized, this crosstalk between the two alleles is mostly mediated by short RNAs which act in trans and establish a transcriptionally silent chromatin state which is meiotically heritable through several generations. Epigenetic states assigned by paramutagenic alleles are occasionally permanent (Coe 1966) and found in all future generations, and some are reversed after few generations (Belele et al. 2013; Brink 1956; Goettel and Messing 2013; Gross and Hollick 2007; Hollick and Chandler 1998; Hollick et al. 1995; Styles and Brink 1969), as they show less then 100% heritability (Hollick 2017).

While not demonstrated, the study on the INS VNTR alleles likely represent the first paramutation-like example in humans, where the protective class III allele modifies in trans the class I risk allele on the same locus and determines offspring phenotypes (Bennett et al. 1997). Similarly, the first paramutation-like example in mice was reported by the group of Paul Soloway at Cornell University (US) (Herman et al. 2003), which, by using the Rasgrf1 and the Igf2r alleles, respectively, maternally and paternally imprinted, and replacing the Rasgrf1 imprinting control region with the one on the Igf2r allele, showed that the Rasgrf1 maintained its paternal expression, and transmission of the mutated paternal allele to the offspring was also able to methylate and activate the normally silent (imprinted) maternal allele, which remained stable through generations and independently from the presence of the mutated allele. While involving complex genetic manipulations (such as the transfer of oppositely imprinted sequences between loci) to initiate the trans-allelic interactions, the effects of such interaction continue in the wild-type progeny thus suggesting that they constitute normally occurring genomic events. Also, these findings recapitulate two tenets of paramutation: trans-allele interaction; and stability through meiosis (Herman et al. 2003). These studies are purely descriptive and fail in identifying the molecular determinants of trans-allele interaction.

An attempt in this direction is instead provided by the study from Rassoulzadegan et al., which showed that Kit mutation perturbs the Kit/KitL signaling pathway and induces deregulation of several regulatory RNAs (mostly miRNAs), which—by targeting the wild-type Kit mRNA—are sufficient to recapitulate the offspring phenotype when microinjected into wild-type fertilized oocytes (Rassoulzadegan et al. 2006). Interestingly, not only sperm miRNAs, but also brain miRNAs and random RNA sequences resembling the wild-type Kit mRNA are sufficient to reproduce the phenotype. Although the origin of the regulatory RNAs is unknown and genetic effects are not completely ruled out, the reported findings are intriguing as they constitute the first example of RNA-based epigenetic inheritance in mice—now considered the most likely mechanism of epigenetic inheritance—and, while not fully understood, suggest a mechanism which recalls paramutation in maize (Soloway 2006). The same group has further shown that zygotic microinjection of specific miRNAs targeting genes involved in key developmental processes leads to offspring growth disturbances (miR124 → Sox9) (Grandjean et al. 2009) and cardiac hypertrophy (miR1 → Cdk9) (Wagner et al. 2008).

As just mentioned, small non-coding RNAs have emerged as the best candidates to explain heritable epigenetic regulation of gene expression and currently represent the most likely molecular underpinning of acquired epigenetic inheritance as well (Chen et al. 2016; de Castro Barbosa et al. 2016; Grandjean et al. 2015; Sharma et al. 2016; Yuan et al. 2016) (Zhang et al. 2018). Small RNAs, including miRNAs, piwi RNAs, tRNA-derived small RNAs (tsRNAs) and repeat-associated small RNAs, do indeed trigger heritable gene silencing (Alcazar et al. 2008; Grishok et al. 2000; Heard and Martienssen 2014) and have the potential to influence developmental and adult phenotypic trajectories (Conine et al. 2018; Yuan et al. 2016). Sperm loaded RNAs acquired during the epididymal transit are—for example—essential for proper embryonic development, and by targeting a sensitive gene family in pre-implantation embryos (Conine et al. 2018), affect adult phenotypic trajectories.

Altogether, these findings are in line with many features of paramutations and show that (1) Regulatory sequences can function ectopically (the wild-type Rasgrf1 allele controls the expression of the mutated one); (2) Paramutation-like phenotypes are heritable and stable through generations independently from the presence of the paramutagenic allele; and (3) Paramutation-like phenomena can be artificially induced supporting its sequence-driven nature.

Although paramutations—defined as RNA-based trans-homologous epigenetic modulation of gene expression—are unlikely to globally explain indirect genetic effects in mammals, the concept of trans-acting mechanisms which, triggered by mutated alleles, can induce trans-acting stable and heritable epigenetic reprogramming at other alleles of the same or—most likely—different loci and multigenerational phenotype transfer, is indeed interesting and worth deeper investigation.

The examples of indirect genetic effects presented in this review already constitute a compelling catalog, as they encompass various complex phenotypes and diverse genetic triggers. The next and critical questions to address will be how common these indirect genetic effects are in mammals, which complex phenotypes they can trigger/stabilize and—most importantly—whether they can reveal at least part of the missing heritability highlighted by genome-wide association studies.

## Conclusions and future perspectives

The main goal of this review was to convey the message that indirect genetic effects—broadly defined as genetically determined (as they are determined by the parental genotype), genotype-independent (as their manifestation is independent from the carried genotype) control of phenotypic variation across generations—may be more common and relevant than actually thought for the pathophysiology and the heritability of complex traits in mammals. Further and deeper study of indirect genetic effects might therefore constitute a strategy to identify at least part of the missing heritability associated with GWAS signals and might help shedding light on complex disease biology and individual disease susceptibility in humans.

A first and necessary step towards unraveling the real potential of indirect genetic effects for human health is to understand how common they really are in mammals, and to identify associated genes and/or gene families and functions. This need calls for the importance for the scientific community to have access to unbiased, comprehensive, systemic and highly controlled datasets. One example of those datasets is the one provided by the efforts of the International Mouse Phenotyping Consortium (IMPC). The IMPC is an international establishment, which aims to generate and systemically phenotype—with tests encompassing a wide range of system areas, including neurological, behavioral, metabolism, cardiovascular, pulmonary, reproductive, sensory and musculoskeletal functions—mouse mutants for every gene in the mouse genome. A combined effort of more than 20 institutions worldwide provides access for the scientific community to comprehensive and standardized mouse phenotypic data for the purpose of identifying human-relevant disease causing genes (Brown and Moore 2012). To date, the IMPC has generated 6255 mutant lines and 5861 of them have been phenotyped. Critically, already 360 IMPC lines (40%) have phenotypic similarity with 889 human disease genes, and the majority (78%) of these lines are the first reported mouse models for the associated human diseases (Meehan et al. 2017). Beyond this, the IMPC resource has been used in more than 2000 published studies so far, and has led to the identification of essential genes for mouse viability (Cacheiro et al. 2020), as well as candidate genes for metabolic homeostasis (Rozman et al. 2018), eye development (Moore et al. 2018), auditory dysfunction (Bowl et al. 2017) and sexual dimorphism (Karp et al. 2017).

These results reinforce the concept that phenotypic data from mouse models hold potential critical relevance to direct clinical studies in humans. Such large-scale and multidimensional gene-phenotype datasets are of help to shed new light on our understanding of mammalian gene function and disease association. Implementing strategies to include systematic analysis of indirect genetic effects into large-scale mouse phenotyping efforts (such as the one exemplified by the IMPC) will add a new dimension to gene function (gene-dependent/genotype-independent), shed light on parental contribution to offspring phenotypic trajectories and hopefully boost discoveries on human genetics and the relevance of human genetic variation for individual susceptibility to complex diseases, such as diabetes, obesity and cancer.

## References

[CR1] Alcazar RM, Lin R, Fire AZ (2008). Transmission dynamics of heritable silencing induced by double-stranded RNA in *Caenorhabditis elegans*. Genetics.

[CR2] Anway MD, Cupp AS, Uzumcu M, Skinner MK (2005). Epigenetic transgenerational actions of endocrine disruptors and male fertility. Science.

[CR3] Belele CL, Sidorenko L, Stam M, Bader R, Arteaga-Vazquez MA, Chandler VL (2013). Specific tandem repeats are sufficient for paramutation-induced trans-generational silencing. PLoS Genet.

[CR4] Bennett N, Wood L, Rogers S (1997). Teaching through play: teachers' thinking and classroom practise.

[CR5] Bijma P (2014). The quantitative genetics of indirect genetic effects: a selective review of modelling issues. Heredity.

[CR6] Blewitt ME, Vickaryous NK, Hemley SJ, Ashe A, Bruxner TJ, Preis JI, Arkell R, Whitelaw E (2005). An N-ethyl-N-nitrosourea screen for genes involved in variegation in the mouse. Proc Natl Acad Sci USA.

[CR7] Bonduriansky R, Day T (2018). Extended heredity: a new understanding of inheritance and evolution.

[CR8] Bowl MR, Simon MM, Ingham NJ, Greenaway S, Santos L, Cater H, Taylor S, Mason J, Kurbatova N, Pearson S, Bower LR, Clary DA, Meziane H, Reilly P, Minowa O, Kelsey L, Tocchini-Valentini GP, Gao X, Bradley A, Skarnes WC, Moore M, Beaudet AL, Justice MJ, Seavitt J, Dickinson ME, Wurst W, de Angelis MH, Herault Y, Wakana S, Nutter LMJ, Flenniken AM, McKerlie C, Murray SA, Svenson KL, Braun RE, West DB, Lloyd KCK, Adams DJ, White J, Karp N, Flicek P, Smedley D, Meehan TF, Parkinson HE, Teboul LM, Wells S, Steel KP, Mallon AM, Brown SDM (2017). A large scale hearing loss screen reveals an extensive unexplored genetic landscape for auditory dysfunction. Nat Commun.

[CR9] Brink RA (1956). A genetic change associated with the R locus in maize which is directed and potentially reversible. Genetics.

[CR10] Brink RA, Brown DF, Kermicle J, Weyers WH (1960). Locus dependence of the paramutant R phenotype in maize. Genetics.

[CR11] Brown DF, Brink RA (1960). Paramutagenic action of paramutant R and R alleles in maize. Genetics.

[CR12] Brown SD, Moore MW (2012). The international mouse phenotyping consortium: past and future perspectives on mouse phenotyping. Mamm Genome.

[CR13] Buniello A, MacArthur JAL, Cerezo M, Harris LW, Hayhurst J, Malangone C, McMahon A, Morales J, Mountjoy E, Sollis E, Suveges D, Vrousgou O, Whetzel PL, Amode R, Guillen JA, Riat HS, Trevanion SJ, Hall P, Junkins H, Flicek P, Burdett T, Hindorff LA, Cunningham F, Parkinson H (2019). The NHGRI-EBI GWAS catalog of published genome-wide association studies, targeted arrays and summary statistics 2019. Nucleic Acids Res.

[CR14] Cacheiro P, Munoz-Fuentes V, Murray SA, Dickinson ME, Bucan M, Nutter LMJ, Peterson KA, Haselimashhadi H, Flenniken AM, Morgan H, Westerberg H, Konopka T, Hsu CW, Christiansen A, Lanza DG, Beaudet AL, Heaney JD, Fuchs H, Gailus-Durner V, Sorg T, Prochazka J, Novosadova V, Lelliott CJ, Wardle-Jones H, Wells S, Teboul L, Cater H, Stewart M, Hough T, Wurst W, Sedlacek R, Adams DJ, Seavitt JR, Tocchini-Valentini G, Mammano F, Braun RE, McKerlie C, Herault Y, de Angelis MH, Mallon AM, Lloyd KCK, Brown SDM, Parkinson H, Meehan TF, Smedley D (2020). Human and mouse essentiality screens as a resource for disease gene discovery. Nat Commun.

[CR15] Carouge D, Blanc V, Knoblaugh SE, Hunter RJ, Davidson NO, Nadeau JH (2016). Parent-of-origin effects of A1CF and AGO2 on testicular germ-cell tumors, testicular abnormalities, and fertilization bias. Proc Natl Acad Sci USA.

[CR16] Chen Q, Yan M, Cao Z, Li X, Zhang Y, Shi J, Feng GH, Peng H, Zhang X, Zhang Y, Qian J, Duan E, Zhai Q, Zhou Q (2016). Sperm tsRNAs contribute to intergenerational inheritance of an acquired metabolic disorder. Science.

[CR17] Chong S, Vickaryous N, Ashe A, Zamudio N, Youngson N, Hemley S, Stopka T, Skoultchi A, Matthews J, Scott HS, de Kretser D, O'Bryan M, Blewitt M, Whitelaw E (2007). Modifiers of epigenetic reprogramming show paternal effects in the mouse. Nat Genet.

[CR18] Coe EH (1959). A regular and continuing conversion-type phenomenon at the B locus in maize. Proc Natl Acad Sci USA.

[CR19] Coe EH (1966). The properties, origin, and mechanism of conversion-type inheritance at the B locus in maize. Genetics.

[CR20] Conine CC, Sun F, Song L, Rivera-Perez JA, Rando OJ (2018). Small RNAs gained during epididymal transit of sperm are essential for embryonic development in mice. Dev Cell.

[CR21] Cordell HJ (2009). Detecting gene-gene interactions that underlie human diseases. Nat Rev Genet.

[CR22] Costanzo M, VanderSluis B, Koch EN, Baryshnikova A, Pons C, Tan G, Wang W, Usaj M, Hanchard J, Lee SD, Pelechano V, Styles EB, Billmann M, van Leeuwen J, van Dyk N, Lin ZY, Kuzmin E, Nelson J, Piotrowski JS, Srikumar T, Bahr S, Chen Y, Deshpande R, Kurat CF, Li SC, Li Z, Usaj MM, Okada H, Pascoe N, San Luis BJ, Sharifpoor S, Shuteriqi E, Simpkins SW, Snider J, Suresh HG, Tan Y, Zhu H, Malod-Dognin N, Janjic V, Przulj N, Troyanskaya OG, Stagljar I, Xia T, Ohya Y, Gingras AC, Raught B, Boutros M, Steinmetz LM, Moore CL, Rosebrock AP, Caudy AA, Myers CL, Andrews B, Boone C (2016). A global genetic interaction network maps a wiring diagram of cellular function. Science.

[CR23] Dalgaard K, Landgraf K, Heyne S, Lempradl A, Longinotto J, Gossens K, Ruf M, Orthofer M, Strogantsev R, Selvaraj M, Lu TT, Casas E, Teperino R, Surani MA, Zvetkova I, Rimmington D, Tung YC, Lam B, Larder R, Yeo GS, O'Rahilly S, Vavouri T, Whitelaw E, Penninger JM, Jenuwein T, Cheung CL, Ferguson-Smith AC, Coll AP, Korner A, Pospisilik JA (2016). Trim28 haploinsufficiency triggers bi-stable epigenetic obesity. Cell.

[CR24] Darr J, Tomar A, Lassi M, Gerlini R, Berti L, Hering A, Scheid F, Hrabe de Angelis M, Witting M, Teperino R (2020). iTAG-RNA isolates cell-specific transcriptional responses to environmental stimuli and identifies an RNA-based endocrine axis. Cell Rep.

[CR25] David I, Canario L, Combes S, Demars J (2019). Intergenerational transmission of characters through genetics, epigenetics, microbiota, and learning in livestock. Front Genet.

[CR26] de Castro BT, Ingerslev LR, Alm PS, Versteyhe S, Massart J, Rasmussen M, Donkin I, Sjogren R, Mudry JM, Vetterli L, Gupta S, Krook A, Zierath JR, Barres R (2016). High-fat diet reprograms the epigenome of rat spermatozoa and transgenerationally affects metabolism of the offspring. Mol Metab.

[CR27] Dias BG, Ressler KJ (2014). Parental olfactory experience influences behavior and neural structure in subsequent generations. Nat Neurosci.

[CR28] Duncan L, Shen H, Gelaye B, Meijsen J, Ressler K, Feldman M, Peterson R, Domingue B (2019). Analysis of polygenic risk score usage and performance in diverse human populations. Nat Commun.

[CR29] Fang G, Wang W, Paunic V, Heydari H, Costanzo M, Liu X, Liu X, VanderSluis B, Oately B, Steinbach M, Van Ness B, Schadt EE, Pankratz ND, Boone C, Kumar V, Myers CL (2019). Discovering genetic interactions bridging pathways in genome-wide association studies. Nat Commun.

[CR30] Franberg M, Gertow K, Hamsten A, Consortium P, Lagergren J, Sennblad B (2015). Discovering genetic interactions in large-scale association studies by stage-wise likelihood ratio tests. PLoS Genet.

[CR31] Gayon J (2016). From mendel to epigenetics: history of genetics. C R Biol.

[CR32] Goettel W, Messing J (2013). Paramutagenicity of a p1 epiallele in maize. Theor Appl Genet.

[CR34] Grandjean V, Gounon P, Wagner N, Martin L, Wagner KD, Bernex F, Cuzin F, Rassoulzadegan M (2009). The miR-124-Sox9 paramutation: RNA-mediated epigenetic control of embryonic and adult growth. Development.

[CR33] Grandjean V, Fourre S, De Abreu DA, Derieppe MA, Remy JJ, Rassoulzadegan M (2015). RNA-mediated paternal heredity of diet-induced obesity and metabolic disorders. Sci Rep.

[CR35] Grishok A, Tabara H, Mello CC (2000). Genetic requirements for inheritance of RNAi in *C. elegans*. Science.

[CR36] Gross SM, Hollick JB (2007). Multiple trans-sensing interactions affect meiotically heritable epigenetic states at the maize pl1 locus. Genetics.

[CR37] Hauser MT, Aufsatz W, Jonak C, Luschnig C (2011). Transgenerational epigenetic inheritance in plants. Biochim Biophys Acta.

[CR38] Heard E, Martienssen RA (2014). Transgenerational epigenetic inheritance: myths and mechanisms. Cell.

[CR39] Herman H, Lu M, Anggraini M, Sikora A, Chang Y, Yoon BJ, Soloway PD (2003). Trans allele methylation and paramutation-like effects in mice. Nat Genet.

[CR40] Hill WG, Goddard ME, Visscher PM (2008). Data and theory point to mainly additive genetic variance for complex traits. PLoS Genet.

[CR41] Holeski LM, Jander G, Agrawal AA (2012). Transgenerational defense induction and epigenetic inheritance in plants. Trends Ecol Evol.

[CR42] Hollick JB (2017). Paramutation and related phenomena in diverse species. Nat Rev Genet.

[CR43] Hollick JB, Chandler VL (1998). Epigenetic allelic states of a maize transcriptional regulatory locus exhibit overdominant gene action. Genetics.

[CR44] Hollick JB, Patterson GI, Coe EH, Cone KC, Chandler VL (1995). Allelic interactions heritably alter the activity of a metastable maize pl allele. Genetics.

[CR45] Huypens P, Sass S, Wu M, Dyckhoff D, Tschop M, Theis F, Marschall S, Hrabe de Angelis M, Beckers J (2016). Epigenetic germline inheritance of diet-induced obesity and insulin resistance. Nat Genet.

[CR46] Kam-Thong T, Czamara D, Tsuda K, Borgwardt K, Lewis CM, Erhardt-Lehmann A, Hemmer B, Rieckmann P, Daake M, Weber F, Wolf C, Ziegler A, Putz B, Holsboer F, Scholkopf B, Muller-Myhsok B (2011). EPIBLASTER-fast exhaustive two-locus epistasis detection strategy using graphical processing units. Eur J Hum Genet.

[CR47] Karp NA, Mason J, Beaudet AL, Benjamini Y, Bower L, Braun RE, Brown SDM, Chesler EJ, Dickinson ME, Flenniken AM, Fuchs H, Angelis MH, Gao X, Guo S, Greenaway S, Heller R, Herault Y, Justice MJ, Kurbatova N, Lelliott CJ, Lloyd KCK, Mallon AM, Mank JE, Masuya H, McKerlie C, Meehan TF, Mott RF, Murray SA, Parkinson H, Ramirez-Solis R, Santos L, Seavitt JR, Smedley D, Sorg T, Speak AO, Steel KP, Svenson KL, Wakana S, West D, Wells S, Westerberg H, Yaacoby S, White JK (2017). Prevalence of sexual dimorphism in mammalian phenotypic traits. Nat Commun.

[CR48] Kong A, Thorleifsson G, Frigge ML, Vilhjalmsson BJ, Young AI, Thorgeirsson TE, Benonisdottir S, Oddsson A, Halldorsson BV, Masson G, Gudbjartsson DF, Helgason A, Bjornsdottir G, Thorsteinsdottir U, Stefansson K (2018). The nature of nurture: effects of parental genotypes. Science.

[CR49] Lam MY, Heaney JD, Youngren KK, Kawasoe JH, Nadeau JH (2007). Trans-generational epistasis between Dnd1Ter and other modifier genes controls susceptibility to testicular germ cell tumors. Hum Mol Genet.

[CR50] Legoff L, D’Cruz SC, Tevosian S, Primig M, Smagulova F (2019). Transgenerational inheritance of environmentally induced epigenetic alterations during mammalian development. Cells.

[CR51] Lesch BJ, Tothova Z, Morgan EA, Liao Z, Bronson RT, Ebert BL, Page DC (2019). Intergenerational epigenetic inheritance of cancer susceptibility in mammals. Elife.

[CR52] Lev I, Toker IA, Mor Y, Nitzan A, Weintraub G, Antonova O, Bhonkar O, Ben Shushan I, Seroussi U, Claycomb JM, Anava S, Gingold H, Zaidel-Bar R, Rechavi O (2019). Germ granules govern small RNA inheritance. Curr Biol.

[CR53] Lewis CM, Vassos E (2020). Polygenic risk scores: from research tools to clinical instruments. Genome Med.

[CR54] Li Y, Cho H, Wang F, Canela-Xandri O, Luo C, Rawlik K, Archacki S, Xu C, Tenesa A, Chen Q, Wang QK (2020). Statistical and functional studies identify epistasis of cardiovascular risk genomic variants from genome-wide association studies. J Am Heart Assoc.

[CR55] Liberman N, Wang SY, Greer EL (2019). Transgenerational epigenetic inheritance: from phenomena to molecular mechanisms. Curr Opin Neurobiol.

[CR56] Lim JP, Brunet A (2013). Bridging the transgenerational gap with epigenetic memory. Trends Genet.

[CR57] Lopez-Cortegano E, Caballero A (2019). Inferring the nature of missing heritability in human traits using data from the GWAS catalog. Genetics.

[CR58] Mackay TF (2014). Epistasis and quantitative traits: using model organisms to study gene-gene interactions. Nat Rev Genet.

[CR59] Mackay TF (2015). Epistasis for quantitative traits in Drosophila. Methods Mol Biol.

[CR60] Manolio TA, Collins FS, Cox NJ, Goldstein DB, Hindorff LA, Hunter DJ, McCarthy MI, Ramos EM, Cardon LR, Chakravarti A, Cho JH, Guttmacher AE, Kong A, Kruglyak L, Mardis E, Rotimi CN, Slatkin M, Valle D, Whittemore AS, Boehnke M, Clark AG, Eichler EE, Gibson G, Haines JL, Mackay TF, McCarroll SA, Visscher PM (2009). Finding the missing heritability of complex diseases. Nature.

[CR62] Martin MP, Gao X, Lee JH, Nelson GW, Detels R, Goedert JJ, Buchbinder S, Hoots K, Vlahov D, Trowsdale J, Wilson M, O'Brien SJ, Carrington M (2002). Epistatic interaction between KIR3DS1 and HLA-B delays the progression to AIDS. Nat Genet.

[CR61] Martin AR, Kanai M, Kamatani Y, Okada Y, Neale BM, Daly MJ (2019). Clinical use of current polygenic risk scores may exacerbate health disparities. Nat Genet.

[CR63] Meehan TF, Conte N, West DB, Jacobsen JO, Mason J, Warren J, Chen CK, Tudose I, Relac M, Matthews P, Karp N, Santos L, Fiegel T, Ring N, Westerberg H, Greenaway S, Sneddon D, Morgan H, Codner GF, Stewart ME, Brown J, Horner N, Haendel M, Washington N, Mungall CJ, Reynolds CL, Gallegos J, Gailus-Durner V, Sorg T, Pavlovic G, Bower LR, Moore M, Morse I, Gao X, Tocchini-Valentini GP, Obata Y, Cho SY, Seong JK, Seavitt J, Beaudet AL, Dickinson ME, Herault Y, Wurst W, de Angelis MH, Lloyd KCK, Flenniken AM, Nutter LMJ, Newbigging S, McKerlie C, Justice MJ, Murray SA, Svenson KL, Braun RE, White JK, Bradley A, Flicek P, Wells S, Skarnes WC, Adams DJ, Parkinson H, Mallon AM, Brown SDM, Smedley D (2017). Disease model discovery from 3,328 gene knockouts by the international mouse phenotyping consortium. Nat Genet.

[CR64] Moore BA, Leonard BC, Sebbag L, Edwards SG, Cooper A, Imai DM, Straiton E, Santos L, Reilly C, Griffey SM, Bower L, Clary D, Mason J, Roux MJ, Meziane H, Herault Y, McKerlie C, Flenniken AM, Nutter LMJ, Berberovic Z, Owen C, Newbigging S, Adissu H, Eskandarian M, Hsu CW, Kalaga S, Udensi U, Asomugha C, Bohat R, Gallegos JJ, Seavitt JR, Heaney JD, Beaudet AL, Dickinson ME, Justice MJ, Philip V, Kumar V, Svenson KL, Braun RE, Wells S, Cater H, Stewart M, Clementson-Mobbs S, Joynson R, Gao X, Suzuki T, Wakana S, Smedley D, Seong JK, Tocchini-Valentini G, Moore M, Fletcher C, Karp N, Ramirez-Solis R, White JK, de Angelis MH, Wurst W, Thomasy SM, Flicek P, Parkinson H, Brown SDM, Meehan TF, Nishina PM, Murray SA, Krebs MP, Mallon AM, Lloyd KCK, Murphy CJ, Moshiri A (2018). Identification of genes required for eye development by high-throughput screening of mouse knockouts. Commun Biol.

[CR66] Nelson VR, Spiezio SH, Nadeau JH (2010). Transgenerational genetic effects of the paternal Y chromosome on daughters' phenotypes. Epigenomics.

[CR65] Nelson VR, Heaney JD, Tesar PJ, Davidson NO, Nadeau JH (2012). Transgenerational epigenetic effects of the Apobec1 cytidine deaminase deficiency on testicular germ cell tumor susceptibility and embryonic viability. Proc Natl Acad Sci USA.

[CR67] Nilsson E, King SE, McBirney M, Kubsad D, Pappalardo M, Beck D, Sadler-Riggleman I, Skinner MK (2018). Vinclozolin induced epigenetic transgenerational inheritance of pathologies and sperm epimutation biomarkers for specific diseases. PLoS ONE.

[CR68] O'Brien EA, Ensbey KS, Day BW, Baldock PA, Barry G (2020). Direct evidence for transport of RNA from the mouse brain to the germline and offspring. BMC Biol.

[CR69] Oey H, Whitelaw E (2014). On the meaning of the word 'epimutation'. Trends Genet.

[CR70] Opachaloemphan C, Yan H, Leibholz A, Desplan C, Reinberg D (2018). Recent advances in behavioral (epi)genetics in eusocial insects. Annu Rev Genet.

[CR71] Panzeri I, Pospisilik JA (2018). Epigenetic control of variation and stochasticity in metabolic disease. Mol Metab.

[CR72] Perez MF, Lehner B (2019). Intergenerational and transgenerational epigenetic inheritance in animals. Nat Cell Biol.

[CR73] Posner R, Toker IA, Antonova O, Star E, Anava S, Azmon E, Hendricks M, Bracha S, Gingold H, Rechavi O (2019). Neuronal small RNAs control behavior transgenerationally. Cell.

[CR74] Prokopuk L, Stringer JM, White CR, Vossen R, White SJ, Cohen ASA, Gibson WT, Western PS (2018). Loss of maternal EED results in postnatal overgrowth. Clin Epigenetics.

[CR75] Rassoulzadegan M, Grandjean V, Gounon P, Vincent S, Gillot I, Cuzin F (2006). RNA-mediated non-mendelian inheritance of an epigenetic change in the mouse. Nature.

[CR76] Rozman J, Rathkolb B, Oestereicher MA, Schutt C, Ravindranath AC, Leuchtenberger S, Sharma S, Kistler M, Willershauser M, Brommage R, Meehan TF, Mason J, Haselimashhadi H, Consortium I, Hough T, Mallon AM, Wells S, Santos L, Lelliott CJ, White JK, Sorg T, Champy MF, Bower LR, Reynolds CL, Flenniken AM, Murray SA, Nutter LMJ, Svenson KL, West D, Tocchini-Valentini GP, Beaudet AL, Bosch F, Braun RB, Dobbie MS, Gao X, Herault Y, Moshiri A, Moore BA, Kent Lloyd KC, McKerlie C, Masuya H, Tanaka N, Flicek P, Parkinson HE, Sedlacek R, Seong JK, Wang CL, Moore M, Brown SD, Tschop MH, Wurst W, Klingenspor M, Wolf E, Beckers J, Machicao F, Peter A, Staiger H, Haring HU, Grallert H, Campillos M, Maier H, Fuchs H, Gailus-Durner V, Werner T, Hrabe de Angelis M (2018) Identification of genetic elements in metabolism by high-throughput mouse phenotyping. Nat Commun 9:288 10.1038/s41467-017-01995-2 PMC5773596 29348434

[CR77] Sabour D, Scholer HR (2012). Reprogramming and the mammalian germline: the Weismann barrier revisited. Curr Opin Cell Biol.

[CR78] Sackton TB, Hartl DL (2016). Genotypic context and epistasis in individuals and populations. Cell.

[CR79] Sharma U, Conine CC, Shea JM, Boskovic A, Derr AG, Bing XY, Belleannee C, Kucukural A, Serra RW, Sun F, Song L, Carone BR, Ricci EP, Li XZ, Fauquier L, Moore MJ, Sullivan R, Mello CC, Garber M, Rando OJ (2016). Biogenesis and function of tRNA fragments during sperm maturation and fertilization in mammals. Science.

[CR80] Sharma U, Sun F, Conine CC, Reichholf B, Kukreja S, Herzog VA, Ameres SL, Rando OJ (2018). Small RNAs are trafficked from the epididymis to developing mammalian sperm. Dev Cell.

[CR81] Sidorenko LV, Peterson T (2001). Transgene-induced silencing identifies sequences involved in the establishment of paramutation of the maize p1 gene. Plant Cell.

[CR82] Siklenka K, Erkek S, Godmann M, Lambrot R, McGraw S, Lafleur C, Cohen T, Xia J, Suderman M, Hallett M, Trasler J, Peters AH, Kimmins S (2015). Disruption of histone methylation in developing sperm impairs offspring health transgenerationally. Science.

[CR83] Skvortsova K, Iovino N, Bogdanovic O (2018). Functions and mechanisms of epigenetic inheritance in animals. Nat Rev Mol Cell Biol.

[CR84] Soloway PD (2006). Genetics: paramutable possibilities. Nature.

[CR85] Stringer JM, Forster SC, Qu Z, Prokopuk L, O'Bryan MK, Gardner DK, White SJ, Adelson D, Western PS (2018). Reduced PRC2 function alters male germline epigenetic programming and paternal inheritance. BMC Biol.

[CR86] Styles ED, Brink RA (1969). The metastable nature of paramutable R alleles in maize. IV. Parallel enhancement of R action in heterozygotes with r and in hemizygotes. Genetics.

[CR87] Tam V, Patel N, Turcotte M, Bosse Y, Pare G, Meyre D (2019). Benefits and limitations of genome-wide association studies. Nat Rev Genet.

[CR88] Trerotola M, Relli V, Simeone P, Alberti S (2015). Epigenetic inheritance and the missing heritability. Hum Genomics.

[CR89] Tyler A, Mahoney JM, Carter GW (2020). Genetic interactions affect lung function in patients with systemic sclerosis. G3.

[CR90] Van Steen K, Moore JH (2019). How to increase our belief in discovered statistical interactions via large-scale association studies?. Hum Genet.

[CR91] Wagner KD, Wagner N, Ghanbarian H, Grandjean V, Gounon P, Cuzin F, Rassoulzadegan M (2008). RNA induction and inheritance of epigenetic cardiac hypertrophy in the mouse. Dev Cell.

[CR92] Wang X, Elston RC, Zhu X (2010). The meaning of interaction. Hum Hered.

[CR93] Weismann A (1893) The germ-plasm: a theory of heredity. Translated by W. Newton Parker and Harriet Rönnfeldt, Scribner, New York

[CR94] Wolf JB, Brodie Iii ED, Cheverud JM, Moore AJ, Wade MJ (1998). Evolutionary consequences of indirect genetic effects. Trends Ecol Evol.

[CR95] Yazbek SN, Spiezio SH, Nadeau JH, Buchner DA (2010). Ancestral paternal genotype controls body weight and food intake for multiple generations. Hum Mol Genet.

[CR96] Young AI (2019). Solving the missing heritability problem. PLoS Genet.

[CR97] Yuan S, Schuster A, Tang C, Yu T, Ortogero N, Bao J, Zheng H, Yan W (2016). Sperm-borne miRNAs and endo-siRNAs are important for fertilization and preimplantation embryonic development. Development.

[CR98] Zhang F, Boerwinkle E, Xiong M (2014). Epistasis analysis for quantitative traits by functional regression model. Genome Res.

[CR99] Zhang YF, Zhang XD, Shi JC, Tuorto F, Li X, Liu YS, Liebers R, Zhang LW, Qu YC, Qian JJ, Pahima M, Liu Y, Yan MH, Cao ZH, Lei XH, Cao YJ, Peng HY, Liu SC, Wang Y, Zheng HL, Woolsey R, Quilici D, Zhai QW, Li L, Zhou T, Yan W, Lyko F, Zhang Y, Zhou Q, Duan EK, Chen Q (2018). Dnmt2 mediates intergenerational transmission of paternally acquired metabolic disorders through sperm small non-coding RNAs. Nat Cell Biol.

[CR100] Zuk O, Hechter E, Sunyaev SR, Lander ES (2012). The mystery of missing heritability: Genetic interactions create phantom heritability. Proc Natl Acad Sci USA.

